# Chemical Basis of Traditional Medicines and New Potential Applications

**DOI:** 10.1155/2014/723502

**Published:** 2014-12-31

**Authors:** Chunlin Long, Shi-Biao Wu, William C. S. Cho

**Affiliations:** ^1^College of Life and Environmental Sciences, Minzu University of China, Beijing 100081, China; ^2^Kunming Institute of Botany, Chinese Academy of Sciences, Kunming 650201, China; ^3^Department of Biological Sciences, Lehman College, The City University of New York, Bronx, NY 10468, USA; ^4^Department of Clinical Oncology, Queen Elizabeth Hospital, Kowloon, Hong Kong

Traditional medicines such as Chinese medicine, Ayurveda, Unani, and ethnomedicines have globally been practiced by billions of people for many centuries. In the rural areas of developing countries, traditional medicine is often the only accessible and affordable treatment available. Even in developed countries the use of traditional medicine is gaining popularity, where western medicine is generally available. In Asian and African countries, 80% populations depend on traditional medicines for primary health care according to the World Health Organization. A lot of famous pharmaceutical drugs are derived from traditional medicines (e.g., artemisinin from traditional Chinese medicine* Artemisia annua*). Plants, animals, microbes, and minerals used in traditional medicines are enormous. Only the species number of traditionally used medicinal plants was estimated to be between 10,000 and 53,000.

Although studies on traditional medicines have become a popular research trend worldwide, only a very small proportion of traditional medicines had been investigated focusing on their chemical components and biological activities. There are still a huge number of traditional medicinal species which are not investigated chemically. For the tropical plant species, for example, only 1% of them had been screened. As for the polypharmaceuticals with more than two species, especially in traditional Chinese medicine, less chemical studies had been completed. They are being used by millions of people every day. Therefore, further investigations of chemical basis of traditional medicines will be necessary and urgent. It will be also very important in the future to gain a better understanding of the chemical basis of these traditional medicines, demonstrate their activity, understand their mechanism of action, develop new potential applications, and discover new drugs based on the studies of traditional medicines.

We received more than 30 papers after the call for papers was released in October 2013. Finally, only 18 high-quality peer-reviewed papers were included in this special issue. In addition to one review article, 17 papers are original research articles.

The traditional medicinal plants are often selected for pharmacological studies, followed by isolation and identification of their chemical constituents. In this special issue, some plants are famous traditional herbal medicines such as* Panax notoginseng*,* Angelica sinensis*,* Carthamus tinctorius*, and* Oldenlandia diffusa* (*Hedyotis diffusa*). Some are less well-known but important in local ethnic communities (e.g.,* Selaginella moellendorffii*,* Elephantopus scarber*,* Melastoma malabathricum, Dicranostigma leptopodum*, and* Rabdosia japonica* var.* glaucocalyx*). Besides the medicinal plants, other natural medicinal sources, such as Czech propolis, have also been included in this issue.

Whether the processed or prepared medicines (e.g., Chinese traditional patent medicine and Chinese medicinal formula) are mixed with other materials or not demonstrated different biological activities from their original herbs. Their chemical basis and mechanism of action should be revealed. Paeoniae Radix (processed roots of* Paeonia lactiflora*), Fuzheng Fangai pill (composed of* Codonopsis pilosula*,* Astragalus,* and other 4 species), Tianshu capsule (composed of* Ligusticum chuanxiong* and* Gastrodia elata*), and Sihuangxiechai decoction (composed of* Astragalus* and other 15 species) are included in this special issues. The research results pharmacologically supported their customary uses of these traditional medicines.

It is impressive to note that an important traditional medicinal plant, Huangqi (*Astragalus mongholicus* and/or* A. membranaceus*), appeared in 4 papers in this issue (L. Shi et al., Y. Gao et al., S. Liu et al., and X. P. Huang et al.). Both its secondary metabolites and polysaccharide showed multiple pharmacological activities such as immunomodulatory and neuroprotective effects, anti-inflammation, and antivirus. Further studies on Huangqi may endow this traditional medicine with more new potential applications.

*Jatropha* is ethnobotanically and ethnopharmacologically an important group in Euphorbiaceae, including* J. curcas* and* J. gossypiifolia*. To compare with other* Jatropha* species, few studies have isolated chemical compounds from* J. gossypiifolia*. However, it should be prioritized for bioprospecting.

We believe that this special issue will provide readers with ideas and information in the fields of traditional medicines. Because of the great diversity of traditional medicines, the chemical basis and biological activity should be massively investigated in next decades so as to examine the safety, action mechanisms, new applications, and development potentials of traditional medicines.

*Chunlin  Long* *Chunlin  Long* *Shi-Biao  Wu* *Shi-Biao  Wu* *William  C.  S.  Cho* *William  C.  S.  Cho*

